# Sensitivity Enhancement of Dynamic Full-Field Optical Coherence Tomography Using Ratio-Free Detection and Partial-Field Illumination for Retinal Organoid Imaging

**DOI:** 10.3390/bioengineering13070716

**Published:** 2026-06-23

**Authors:** Tual Monfort

**Affiliations:** Institut de la Vision, Sorbonne Université, INSERM, CNRS, 17 rue Moreau, F-75012 Paris, France; tual.monfort@inserm.fr

**Keywords:** dynamic full-field optical coherence tomography, time domain OCT, partial-field illumination, ratio-free detection, label-free imaging, live-cell imaging

## Abstract

Time-domain dynamic full-field optical coherence tomography (D-FFOCT) is a powerful label-free imaging modality that enables functional visualization of cellular activity in living tissues with subcellular resolution. However, its sensitivity remains a major limitation for imaging highly scattering three-dimensional (3D) biological models such as retinal organoids, where incoherent background and inefficient optical flux distribution reduce dynamic contrast and limit imaging depth. In this work, we introduce a ratio-free optical configuration for time-domain D-FFOCT that enables continuous tuning of the sample-to-reference field ratio while minimizing photon losses and suppressing parasitic reflections. This polarization-based architecture allows optimal redistribution of optical flux according to sample scattering conditions and improves sensitivity under both power-limited and dose-limited conditions. Compared with conventional non-polarizing beam splitter configurations, the proposed approach provides a $\sqrt{2}$-fold (3 dB) sensitivity improvement through optical optimization alone. In addition, we investigate for the first time the use of partial-field illumination (PFI) in time-domain D-FFOCT to reduce incoherent background arising from multiple scattering. In retinal organoids imaged at 120 μm depth, PFI yields up to a 14.5-fold (23.2 dB) increase in dynamic signal sensitivity, while preserving functional contrast. When combined, ratio-free detection and PFI provide a cumulative sensitivity improvement of 20.5-fold (26.2 dB). These gains enable improved cellular-scale visualization in retinal organoids, including cell-resolved imaging within rosette regions, as well as improved detection of intracellular dynamics in Müller glial cell cultures. This work establishes a practical framework for sensitivity optimization in D-FFOCT and expands its potential for functional imaging, disease modeling, and live-cell monitoring in complex biological systems.

## 1. Introduction

Three-dimensional (3D) human cell cultures, organoids, and tissue explants have become essential models for studying human development, disease mechanisms, and therapeutic responses [1,2,3]. Compared with conventional two-dimensional (2D) cultures, these systems better reproduce the structural organization, cellular heterogeneity, and microenvironment of native tissues, making them highly relevant for drug screening, disease modeling, and reducing reliance on animal experimentation [4,5]. In particular, organ-on-a-chip engineering and organoid-based systems have significantly expanded the possibilities for modeling complex physiological environments [1], while advanced imaging approaches have further improved the characterization of these models [2]. In ophthalmology, retinal organoids derived from human induced pluripotent stem cells have emerged as particularly valuable models for investigating retinal development, neurodegeneration, inherited retinal diseases, and photoreceptor physiology [6,7].

Despite their biological relevance, imaging complex 3D models in a label-free configuration remains challenging. Their thickness often exceeds many times the light transport mean free path, resulting in strong multiple scattering, reduced penetration depth, and poor image contrast compared with 2D cultures [7]. Conventional fluorescence microscopy can provide high specificity, and fluorescence nanoscopy has considerably improved subcellular imaging capabilities in cell biology [8]; however, these approaches often require exogenous markers that may alter cellular physiology, limit long-term observations, or become incompatible with clinical translation [9,10,11,12]. Label-free imaging approaches are therefore highly desirable for continuous live-cell monitoring and functional characterization of biological samples [4,5,10].

Beyond structural imaging, recent advances in live-cell microscopy have highlighted the importance of measuring temporal cellular dynamics as functional biomarkers of tissue physiology [11,13,14]. Processes such as proliferation, migration, differentiation, apoptosis, and intracellular transport reflect metabolic activity and cellular health, and their dysregulation is associated with numerous pathological conditions including cancer [9,15,16], autoimmune diseases [17], neurodegeneration [18], and chronic inflammation [19]. Functional imaging of these dynamic processes is therefore critical for understanding disease progression and evaluating therapeutic responses in physiologically relevant models [12,20].

Optical coherence tomography (OCT), and particularly time-domain full-field OCT (FFOCT), addresses many of these challenges by providing label-free, high-resolution en face imaging with submicron axial and lateral resolution [21,22,23]. By exploiting interference between backscattered light from the sample and a reference field, FFOCT enables optical sectioning, signal amplification, and phase-sensitive detection without mechanical scanning [22,24]. This configuration is especially well suited for high-numerical-aperture objectives and has demonstrated sub-cellular-scale imaging in both biological tissues and retinal models [21,24,25].

More recently, dynamic OCT and time-domain dynamic full-field OCT (D-FFOCT) have extended this approach from purely structural imaging toward functional imaging of living systems [26,27]. Rather than relying solely on static reflectivity contrast, D-FFOCT analyzes temporal fluctuations of the interferometric signal to reveal intracellular motion and metabolic activity within dense biological environments [24,27]. Previous studies have validated that these fluctuations arise from active intracellular processes, including organelle transport and cytoskeleton-regulated dynamics, with evidence implicating mitochondria and other intracellular structures as important contributors to the measured signal [28,29]. This dynamic contrast has been shown to correlate with cellular activity and can provide a degree of cell-type specificity [30], offering complementary information to conventional structural FFOCT [24,27,31]. The adaptation of D-FFOCT to incubators and to commercial microscope platforms has further demonstrated its relevance for longitudinal monitoring of organoids, explants, and live-cell cultures [7].

However, sensitivity remains one of the major limitations of D-FFOCT, particularly for highly scattering 3D samples. Current systems typically rely on a 50/50 non-polarizing beam splitter (NPBS), which inherently wastes approximately 75% of the available optical power, including half of the useful OCT signal [27,32]. In addition, incoherent parasitic reflections from cube beam splitters and multiply scattered background light from the sample occupy a significant fraction of the detector dynamic range, directly reducing sensitivity [7]. Although increasing the optical power could, in principle, compensate for these losses, practical limitations arise from the spatially incoherent nature of the illumination and its Köhler coupling to the microscope objective—particularly in long-arm interferometers compatible with commercial systems [7]. In practice, phase stability requires the use of cube beam splitters, which introduce non-negligible incoherent parasitic reflections [7]. Consequently, increasing the source power or altering the splitting ratio also amplifies these parasitic signals, thereby limiting the net gain achievable through power scaling alone.

Furthermore, the reference field is commonly attenuated using neutral density filters or partially reflective mirrors to balance the interferometric signal with the incoherent background [27,32]. While effective under specific conditions, this strategy introduces substantial photon loss and limits adaptability across different imaging depths and sample scattering conditions. Alternative light-efficient OCT configurations have been proposed, including dark-field full-field OCT and optimized Fourier-domain architectures [32,33,34,35], but they are not directly compatible with standard high-numerical-aperture FFOCT implementations and rely on different OCT modalities.

In this work, we revisit a polarization-based optical configuration originally introduced by Beaurepaire et al. [22] and apply it to D-FFOCT. This ratio-free architecture enables continuous tuning of the sample-to-reference field ratio while minimizing photon losses and strongly suppressing parasitic reflections. In parallel, we investigate the impact of partial-field illumination (PFI), previously shown to improve retinal image contrast by reducing multiply scattered light [36], in D-FFOCT to reduce incoherent background arising from multiple scattering. Together, these two approaches provide a practical strategy for sensitivity optimization under both power-limited and dose-limited conditions.

To our knowledge, this is the first study demonstrating partial-field illumination in time-domain D-FFOCT and the first implementation of ratio-free polarization detection for D-FFOCT.

We demonstrate that ratio-free detection improves sensitivity by $\sqrt{2}$ (3 dB) compared with conventional NPBS configurations, while partial-field illumination provides up to a 14.5-fold (23.2 dB) increase in dynamic signal sensitivity in retinal organoids at 120 μm depth. Combined, these approaches yield a cumulative improvement of 20.5-fold (26.2 dB), enabling improved cellular-scale visualization in retinal organoids, including cell-resolved imaging within rosette regions, as well as improved detection of intracellular dynamics in Müller glial cell cultures. These results establish a new framework for improving D-FFOCT performance and expand its potential for functional imaging, disease modeling, and live-cell monitoring in complex biological systems.

## 2. Results

### 2.1. Signal Model and Sensitivity in D-FFOCT

In time-domain FFOCT, the signal detected on a single camera pixel arises from the superposition of coherent and incoherent contributions. Assuming that the depth of field is smaller than the coherence volume, the detected number of photoelectrons per pixel per frame can be expressed as:

(1) $$N_{tot}=N_{r}+N_{s}+2\sqrt{N_{r}N_{s}}\cos\phi +N_{p}+N_{b},$$

where $N_{r}$, and $N_{s}$ denote the detected reference and coherent sample photons, respectively, $N_{p}$ represents parasitic incoherent reflections originating from optical components, and $N_{b}$ corresponds to the incoherent background generated by multiple scattering within the sample. The phase difference between the reference and sample fields is represented by ϕ.

In practical operation, the camera is used close to its full-well capacity $N_{FWC}$ in order to operate in a shot-noise-limited regime where read noise becomes negligible. Under these conditions,

(2) $$N_{r}+N_{s}+N_{p}+N_{b}\approx N_{FWC}.$$

The relevant quantity is therefore not the absolute interferometric signal itself, but the coherent modulation relative to the detector DC level.

In dynamic FFOCT (D-FFOCT), the useful contrast originates from temporal fluctuations in the coherent interference term. For a time series of *M* statistically independent frames, and independently of the initial phase of the scatterers [25], the signal-to-noise ratio (SNR) can be expressed as:

(3) $$SNR=\frac{2\sqrt{M}\sqrt{N_{r}N_{s}}}{\sqrt{N_{r}+N_{s}+N_{p}+N_{b}}}.$$

In the biologically relevant regime where the coherent sample signal remains much smaller than the reference and incoherent background contributions ($N_{r}+N_{p}+N_{b}\gg N_{s}$), the normalized sensitivity becomes:

(4) $$SNR=\frac{2\sqrt{N_{FWC}\;M\;N_{r}\;N_{s}}}{N_{r}+N_{p}+N_{b}}$$

This expression highlights the central limitation of D-FFOCT sensitivity: the coherent signal is not only constrained by the amount of useful sample photons, but also by the total incoherent DC contribution filling the detector dynamic range. In highly scattering biological samples such as retinal organoids, the multiple-scattering background and parasitic reflections can dominate the detected flux, substantially reducing dynamic contrast.

This sensitivity bottleneck is particularly critical for deep imaging, where weak coherent signals must be detected in the presence of strong incoherent background. Optimizing the distribution of optical flux between the sample and reference arms therefore becomes essential for improving D-FFOCT performance.

### 2.2. Optical Flux Optimization: Ratio-Free D-FFOCT

#### 2.2.1. Conventional NPBS Configuration

In conventional D-FFOCT systems, the interferometer is based on a 50/50 non-polarizing beam splitter (NPBS), which imposes a fixed optical power distribution between the sample and reference arms. For a reference reflectivity $r_{w/Si}^{2}=0.2$ [7], the coherent sample and reference photon counts can be written as:

(5) $$N_{s}^{NPBS}=\frac{1}{4}N_{0}s^{2}\;,\;N_{r}^{NPBS}=\frac{1}{4}N_{0}r_{w/Si}^{2},$$

where $N_{0}$ is the number of photons incident on the interferometer during one exposure, and *s* and $r_{w/Si}$ represent the field reflectivities of the sample and reference, respectively. The quantum efficiency is omitted for simplification purposes.

The incoherent contribution generated by multiply scattered light within the sample is modeled as:

(6) $$N_{b}^{NPBS}=\frac{1}{4}N_{0}\gamma s^{2},$$

where γ is the ratio between incoherent and coherent sample return, typically γ≫1 in highly scattering media. In addition, the NPBS introduces parasitic optical incoherent reflections, modeled as:

(7) $$N_{p}^{NPBS}=\frac{1}{4}N_{0}r_{p}^{2},$$

where $r_{p}^{2}$ represents the effective optical parasitic reflection term.

Substituting these contributions into the SNR expression yields the reference sensitivity:

(8) $${SNR}^{NPBS}=\frac{2sr_{w/Si}\sqrt{N_{FWC}M}}{r_{w/Si}^{2}+\gamma s^{2}+r_{p}^{2}}.$$

This formulation shows that sensitivity is fundamentally limited by three competing contributions: the reference intensity, the incoherent sample background, and parasitic reflections from the optical system. Because the 50/50 beam splitter imposes a fixed ratio between the sample and reference arms, no adaptation is possible when sample scattering conditions vary with depth or biological complexity.

As a result, conventional NPBS-based D-FFOCT can operate far from the theoretical optimum in background-limited samples.

#### 2.2.2. Ratio-Free Polarization Architecture

**Figure 1:**
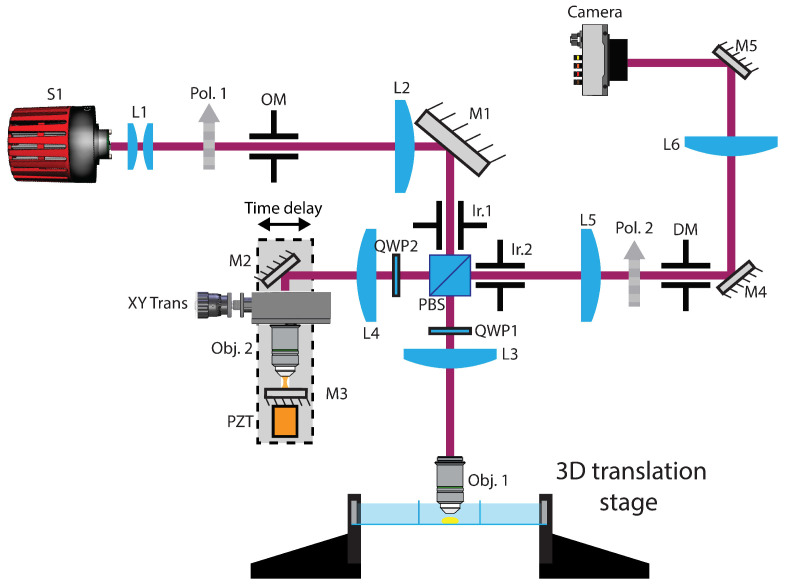
Optical layout of ratio-free dynamic full-field optical coherence tomography (D-FFOCT) system. A mounted light-emitting diode (LED, 810 nm center wavelength, 30 nm bandwidth) was used as an extended spatially incoherent source (S1). A first lens pair (L1) imaged the source onto the illumination mask (OM). A first polarizer (Pol.1), combined with a polarizing beam splitter (PBS), controlled the sample-to-reference optical flux ratio by adjusting the polarization balance between the two interferometer arms. A doublet (L2) relayed the illumination mask to the back focal planes of the microscope objectives (Obj.1 and Obj.2) through the intermediate relay lenses (L3 and L4), forming the Linnik interferometer. The illumination iris (Ir.1), positioned at the focal plane of L2 and conjugated to the sample plane, was used for partial-field illumination (PFI) to reduce incoherent background detected on the camera, generated by multiple scattering. A piezoelectric actuator (PZT) and a linear delay stage introduced dynamic and static phase shifts, respectively. Quarter-wave plates (QWP1 and QWP2) rotated the polarization state after reflection from either the sample or the reference mirror, enabling polarization routing through the PBS and strong suppression of parasitic reflections. A second polarizer (Pol.2) acted as an analyzer to project the orthogonal sample and reference fields onto a common polarization axis before detection by the cMOS camera. This ratio-free polarization architecture enables continuous tuning of the sample-to-reference field ratio, improves detector dynamic range usage, and provides the optical basis for both sensitivity enhancement and partial-field illumination in D-FFOCT.

To overcome these limitations, we implemented a polarization-based ratio-free architecture composed of a polarizer (Pol.1), a polarizing beam splitter (PBS), quarter-wave plates in both interferometer arms, and an analyzer (Pol.2), as shown in Figure 1.

In this configuration, the angle α of Pol.1 controls the distribution of optical power between the sample and reference arms by balancing the horizontal and vertical polarization components of the spatially incoherent source. The analyzer angle β then controls the detected projection of the returned fields.

The detected coherent photon counts become:

(9) $$N_{s}\left(\alpha ,\beta \right)=\frac{N_{0}}{2}s^{2}\sin^{2}\alpha \cos^{2}\beta \;,\;N_{r}\left(\alpha ,\beta \right)=\frac{N_{0}}{2}r^{2}\cos^{2}\alpha \sin^{2}\beta ,$$

where $r^{2}$ is the reflectivity of the reference mirror, typically close to unity. The incoherent sample background remains proportional to the coherent sample return:

(10) $$N_{b}\left(\alpha ,\beta \right)=\gamma \;N_{s}\left(\alpha ,\beta \right),$$

while parasitic optical reflections are strongly suppressed by polarization routing and can be neglected at first order:

(11) $$N_{p}^{PBS}\approx 0.$$

The corresponding interferometric sensitivity becomes:

(12) $${SNR}^{PBS}\left(\alpha ,\beta \right)=\frac{2\sqrt{N_{FWC}MN_{s}\left(\alpha ,\beta \right)N_{r}\left(\alpha ,\beta \right)}}{N_{r}\left(\alpha ,\beta \right)+\gamma N_{s}\left(\alpha ,\beta \right)}.$$

Unlike the conventional NPBS configuration, this ratio-free architecture enables continuous tuning of the sample-to-reference field ratio and direct adaptation to the scattering properties of the biological sample.

**Figure 2:**
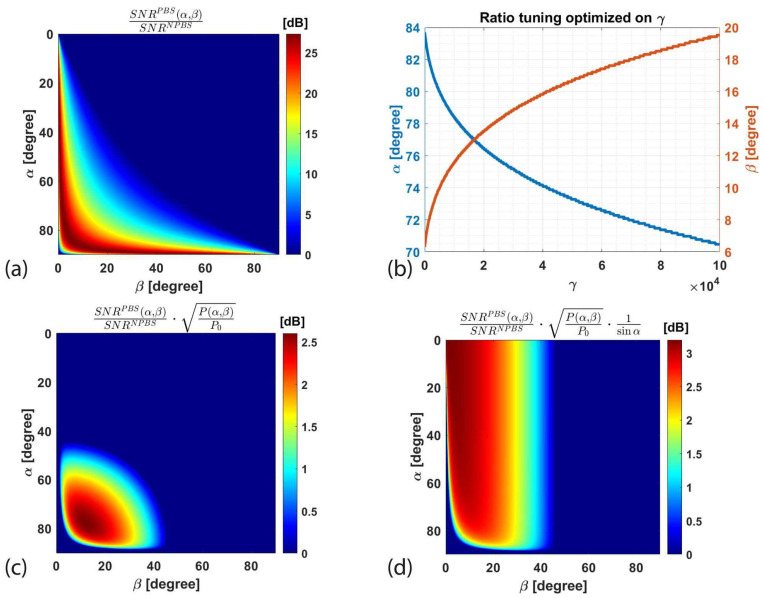
Comparison of signal-to-noise ratio (SNR) between ratio-free PBS and conventional NPBS D-FFOCT configurations. (**a**) Theoretical SNR ratio between the ratio-free polarization-based architecture (PBS) and the conventional 50/50 non-polarizing beam splitter (NPBS) configuration as a function of the polarizer angles *α* (Pol.1) and *β* (Pol.2). The ratio-free configuration provides a substantial sensitivity advantage across a broad range of optical ratios. (**b**) Dependence of optimal SNR on the selected sample-to-reference ratio under increasing incoherent background contributions *γ*, highlighting the importance of adaptive ratio tuning in highly scattering samples. (**c**) SNR improvement obtained under fixed available optical power after including the accumulation gain associated with detector full-well saturation. (**d**) Global experimental sensitivity gain achieved using the ratio-free architecture compared with the conventional NPBS configuration, showing an overall improvement of approximately 3 dB $\sqrt{2}$-fold. These results demonstrate that sensitivity optimization depends critically on optical flux distribution rather than total illumination power alone.

Figure 2a shows the theoretical SNR ratio between the ratio-free PBS architecture and the conventional NPBS configuration. A clear sensitivity advantage is observed across a wide range of configurations, with improvements exceeding 25 dB under ideal optical balancing conditions.

Importantly, the maximum sensitivity strongly depends on the selected values of α and β, as shown in Figure 2b, relative to the amount of incoherent light detected. In background-limited samples, optimal performance is achieved by reducing the sample illumination fraction while maintaining sufficient reference intensity to bring the camera close to saturation.

### 2.3. Sensitivity Gain Mechanisms

#### 2.3.1. Accumulation Gain Under Full-Well Constraint

Because both optical configurations use the same detector, the exposure time required to reach camera saturation depends directly on the total detected DC flux. In the shot-noise-limited regime, the saturation time scales inversely with the detected optical power:

(13) $$t_{sat}\left(\alpha ,\beta \right)\propto \frac{N_{FWC}}{P\left(\alpha ,\beta \right)},$$

where the total detected DC contribution is given by:

(14) $$P\left(\alpha ,\beta \right)\propto \;N_{r}\left(\alpha ,\beta \right)+\gamma N_{s}\left(\alpha ,\beta \right).$$

For a fixed total acquisition time, a shorter saturation time allows a larger number of independent frames to be accumulated. Since D-FFOCT sensitivity scales with the square root of the number of frames, this produces an additional accumulation gain:

(15) $$G_{acc}\left(\alpha ,\beta \right)=\sqrt{\frac{P\left(\alpha ,\beta \right)}{P_{0}}},$$

where $P_{0}$ corresponds to the DC level of the conventional NPBS configuration. For example, if the ratio-free polarization architecture reaches full-well capacity twice as fast as the conventional NPBS setup, twice as many independent frames can be acquired over the same experimental duration, resulting in an additional sensitivity improvement of $\sqrt{2}$.

Figure 2c shows the contribution of this accumulation term to the global SNR comparison. Under fixed available optical power, the ratio-free configuration provides an additional sensitivity improvement of approximately 2.6 dB compared with the classical NPBS architecture.

#### 2.3.2. Dose-Limited Operation

A major advantage of the ratio-free architecture is the decoupling of sample illumination from reference intensity. In the conventional NPBS configuration, increasing the reference field necessarily increases the optical dose delivered to the sample. In contrast, the polarization-based architecture allows these two quantities to be adjusted independently.

Because only a fraction $\sin^{2}\alpha$ of the incident light is directed toward the sample, the optical dose per frame scales as:

(16) $$D\left(\alpha \right)\propto \;N_{0}\sin^{2}\alpha .$$

For a fixed maximum allowable sample dose—particularly important for clinical retinal imaging and long-term live-cell observations—the total incident photon flux can therefore be increased as:

(17) $$N_{dose}\left(\alpha \right)\propto \frac{1}{\sin^{2}\alpha }.$$

This additional available flux increases the coherent interference term, which scales with $\sqrt{N_{dose}}$, resulting in a dose-limited gain factor:

(18) $$G_{dose}\left(\alpha \right)=\frac{1}{\sin\alpha }.$$

Thus, by reducing the fraction of light directed toward the sample while preserving reference intensity, the ratio-free architecture maximizes the information extracted from a constrained photon budget without increasing biological irradiation.

This feature is particularly relevant for retinal applications, where optical dose must remain strictly below safety limits, such as clinical imaging, as well as for long-term imaging of sensitive biological samples such as organoids and stem-cell-derived cultures.

#### 2.3.3. Global Performance

Combining the intrinsic SNR improvement, the accumulation gain, and the dose-limited operation yields the global merit factor:

(19) $$\Gamma \left(\alpha ,\beta \right)=\frac{{SNR}^{PBS}\left(\alpha ,\beta \right)}{{SNR}^{NPBS}}\cdot \frac{1}{\sin\alpha }\cdot \sqrt{\frac{P\left(\alpha ,\beta \right)}{P_{0}}}.$$

This expression summarizes the full benefit of the ratio-free architecture under realistic experimental conditions.

Experimentally, the ratio-free D-FFOCT configuration produces an overall sensitivity improvement of approximately 3 dB compared with the conventional NPBS setup (Figure 2d), corresponding to a $\sqrt{2}$-fold gain obtained purely through optical flux optimization.

The need for brighter spatially incoherent sources therefore becomes particularly relevant, as higher source power can be efficiently exploited in this ratio-free configuration, where parasitic reflections are strongly suppressed through polarization rejection.

This ratio flexibility is especially useful in retinal organoids imaged at depth, where conventional NPBS-based systems rapidly become background-limited.

Beyond the sensitivity gain itself, this architecture provides a major translational advantage for clinical imaging applications: it maximizes usable interferometric contrast under strict optical safety constraints, making it particularly well suited for retinal imaging and future in vivo applications.

### 2.4. Biological Validation in Retinal Organoids and Müller Glial Cells

#### 2.4.1. Ratio-Free D-FFOCT Imaging of Retinal Organoids

To validate the practical impact of the ratio-free architecture, we performed D-FFOCT imaging of human retinal organoids at day 150 of differentiation, corresponding to a previously established and extensively characterized human iPSC-derived retinal organoid model [6], using the optical configuration described in Figure 1. Imaging was performed at a depth of 50 μm using a water-immersion objective of numerical aperture 0.5.

**Figure 3:**
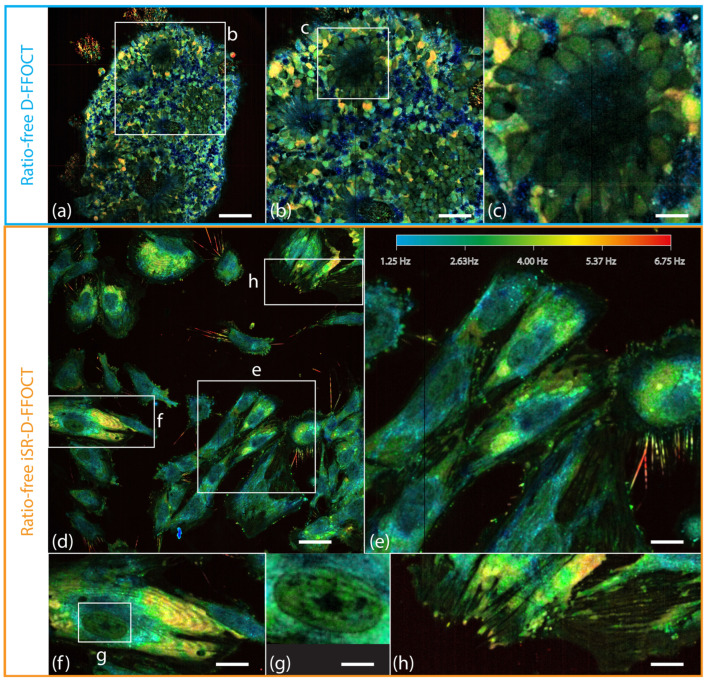
Ratio-free D-FFOCT and interface self-referenced D-FFOCT (iSR-D-FFOCT) imaging of retinal organoids and Müller glial cells. (**a**) Ratio-free D-FFOCT image of a day-150 retinal organoid acquired at 50 μm depth. (**b**) Higher magnification of (**a**), showing improved visualization of cellular boundaries in regions consistent with photoreceptor precursor organization and improved delineation of local cellular organization. (**c**) Zoom-in of (**b**) highlighting a retinal rosette structure, a region that is typically difficult to image due to strong multiple scattering and local disruption of retinal lamination. (**d**) iSR-D-FFOCT image of human induced pluripotent stem cell-derived Müller glial cells cultured in 2D. (**e**) Higher magnification of (**d**) showing several Müller cells with apical microvilli-like structures and bright dynamic intracellular compartments suggestive of highly active membrane-associated or metabolic processes. (**f**) Zoom-in of (**d**) showing interference fringes generated by interference between the plastic–sample interface and the upper membrane–medium interface. (**g**) Higher magnification of (**f**) highlighting a clearly resolved nucleus with visible contour definition and dark low-dynamic structures consistent with heterochromatin organization. (**h**) Zoom-in of (**d**) showing highly dynamic protrusive structures resembling filopodia-like extensions. Hue scales linearly from 1.25 to 6.75 Hz in all images. Scale bars: 80 μm in (**a**), 40 μm in (**b**), 12 μm in (**c**), 50 μm in (**d**), 22 μm in (**e**), 24 μm in (**f**), 10 μm in (**g**), and 20 μm in (**h**).

Despite the relatively moderate numerical aperture, the ratio-free configuration enabled clear visualization of cellular boundaries in regions consistent with photoreceptor precursor organization, based on the established differentiation stage and previously characterized organization of this retinal organoid model [6] (Figure 3a–c). Cellular contours were sufficiently well resolved to allow segmentation of individual cells.

Particularly notable was the visualization of rosette structures, as highlighted in Figure 3c. These regions are difficult to image because local disruption of retinal lamination increases optical aberrations and multiple scattering. The ratio-free configuration enabled clearer structural identification of rosettes and surrounding photoreceptor precursors [6], supporting functional analysis of retinal organization.

Dead or metabolically inactive cells could also be identified through their characteristic saturated, speckled, and blue-shifted dynamic appearance [7]. In the present experiments, this was partly attributed to imaging performed outside the incubator at ambient temperature, illustrating the sensitivity of D-FFOCT dynamic contrast to physiological state and environmental conditions.

#### 2.4.2. Interface Self-Referenced D-FFOCT of Müller Glial Cells

The ratio-free architecture also enables an optimal self-referenced configuration of D-FFOCT, referred to as interface self-referenced D-FFOCT (iSR-D-FFOCT) [37], obtained by setting $\alpha =90^{\circ }$ and $\beta =0^{\circ }$.

In this configuration, interference occurs between the backscattered sample field and the specular reflection generated at the plastic–sample interface (bottom of the multiwell plate), creating an intrinsically stable OCT reference. This self-referenced approach provides strong interferometric contrast while greatly reducing sensitivity to mechanical vibrations and phase instability [37].

Compared with conventional NPBS-based iSR-D-FFOCT, the polarization implementation preserves twice as much useful optical flux originating from the sample (sample field plus reference field), resulting in an additional $\sqrt{2}$-fold (3 dB) sensitivity improvement.

This configuration was applied to 2D cultures of human induced pluripotent stem cell-derived Müller glial cells (Figure 3d–h), previously extensively characterized using immunocytochemical and molecular analyses [38]. Cellular morphology was visualized with high dynamic contrast, including nuclei, microvilli-like apical structures, and highly active protrusive regions resembling filopodia-like extensions.

Figure 3g highlights a particularly well-resolved nucleus with visible contour definition and dark low-dynamic structures consistent with heterochromatin organization. Figure 3h reveals proliferative or protrusive structures with increased dynamic activity at the cellular edge, suggesting active membrane remodeling.

Interestingly, large bright dynamic pockets with characteristic green-yellow hue (≈4–5 Hz) were observed for the first time in 2D Müller cell cultures using iSR-D-FFOCT (Figure 3e). Their biological origin remains under investigation, but their strong dynamic signature suggests highly active intracellular or membrane-associated processes.

Interference fringes were also observed in some cells (Figure 3f), resulting from interference between two specular interfaces: the plastic–sample interface and the upper membrane–medium interface. While such fringes can be reduced using broader spectral bandwidth illumination or higher numerical aperture objectives, they also provide a unique opportunity to probe nanometric membrane deformations and subtle 3D morphological variations.

Together, these results demonstrate that the ratio-free architecture improves both conventional D-FFOCT and self-referenced D-FFOCT, enabling functional imaging of subtle biological structures that remain difficult to detect using standard configurations.

### 2.5. Multi-Scattering Mitigation Using Partial Field Illumination

#### 2.5.1. Partial Field Illumination Strategy

Once optical flux distribution was optimized using the ratio-free architecture, we next addressed the second major limitation of D-FFOCT sensitivity: the incoherent background generated by multiple scattering within the sample.

In retinal organoids imaged at depth, multiply scattered photons increase the detector DC level without contributing to coherent interference, thereby reducing the detectability of weak dynamic signals.

To reduce this background contribution, we implemented partial-field illumination (PFI) using the illumination iris (Ir.1 in Figure 1), thereby restricting the illuminated field of view while preserving interferometric detection. This approach reduces the amount of multiply scattered light reaching the detector at the expense of decreasing the imaging area, effectively improving the coherent-to-incoherent signal ratio.

Exposure times were adjusted to preserve comparable detector filling conditions: 3.08 ms for full-field illumination and 9.85 ms for 8% PFI. At 120 μm depth, comparison of the exposure times required to achieve comparable detector filling under full-field and PFI conditions indicates that more than two-thirds (≈69%) of the detected DC signal was associated with multiple-scattering contributions suppressed by PFI. This observation highlights the dominant contribution of multiple scattering to the detected signal at this depth.

#### 2.5.2. Static and Dynamic Signal Enhancement

**Figure 4:**
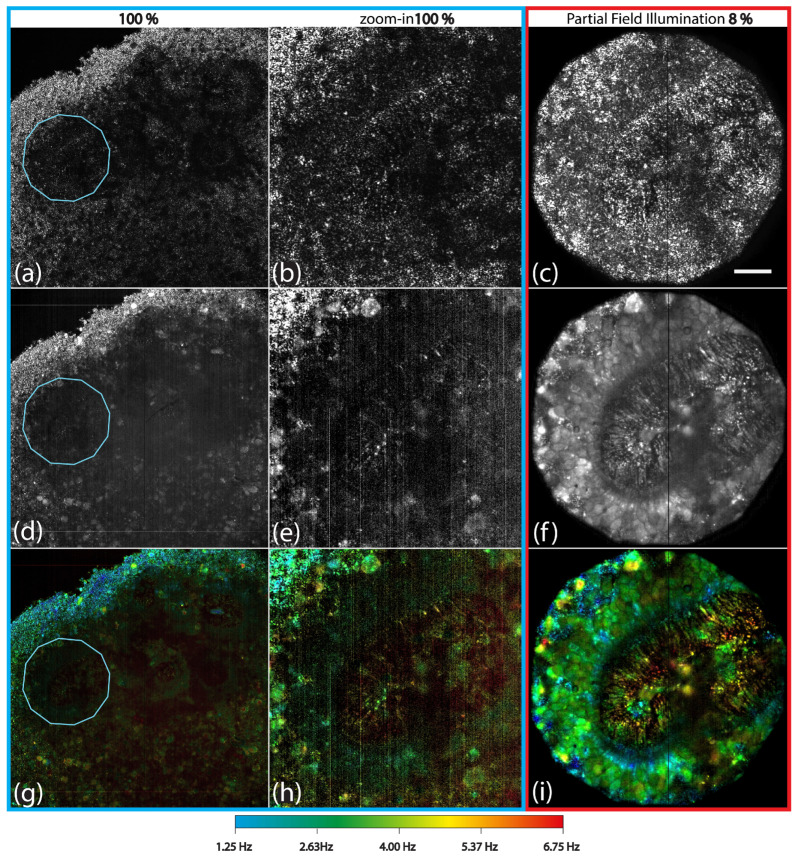
Effect of partial-field illumination (PFI) on static and dynamic D-FFOCT sensitivity in retinal organoids. (**a**–**i**) Static and dynamic D-FFOCT data acquired from a retinal organoid at 120 μm depth using 512 raw interferometric frames acquired at 100 Hz. Exposure times were 3.08 ms for full-field illumination (100%) and 9.85 ms for partial-field illumination (8%), while maintaining a constant reference-to-sample ratio. (**a**–**c**) Static two-phase FFOCT signal. (**d**–**f**) Phase Fluctuation Index (PhFI). (**g**–**i**) Hue–Saturation–Brightness rendering combining the first two moments of the magnitude spectral density and the PhFI. (**a**,**b**,**d**,**e**,**g**,**h**) correspond to conventional full-field illumination (100%), whereas (**c**,**f**,**i**) correspond to 8% partial-field illumination. The blue ROI indicates the effective illuminated field corresponding to the 8% PFI condition. Partial-field illumination substantially improves both structural and dynamic contrast, enabling clear visualization of retinal rosette structures and cellular morphology that remain poorly resolved under conventional illumination. Scale bars: 50 μm in (**a**–**c**) and 25 μm in (**d**–**i**). Hue scales linearly from 1.25 Hz (blue) to 6.75 Hz (red).

Figure 4 compares the effect of PFI on both static and dynamic D-FFOCT signals.

In the conventional full-field configuration, static structural contrast remained partially visible (Figure 4a,b), but dynamic contrast metrics such as the Phase Fluctuation Index (PhFI), mean of the magnitude spectral density (MSD), and standard deviation of MSD showed limited cellular information (Figure 4d,e,g,h). In particular, rosette structures (one of them is circled by a blue ROI in Figure 4a,d,g) and individual cellular boundaries remained poorly resolved because the dynamic signal amplitude was close to the system noise floor.

Under 8% partial-field illumination, both structural and dynamic contrast improved markedly (Figure 4c,f,i). Cellular morphology became clearly visible, and rosette organization could be identified with substantially improved contrast. Dynamic cellular activity was also more readily distinguished, revealing structures that remained undetectable under conventional full-field illumination.

Because D-FFOCT relies on weak temporal fluctuations rather than strong static reflectivity, reducing incoherent background produces a proportionally larger gain in dynamic contrast than in structural imaging.

The improved visualization observed in retinal rosettes further highlights the importance of reducing multiply scattered background for deep D-FFOCT imaging of retinal organoids.

#### 2.5.3. Quantitative Sensitivity Gain

**Figure 5:**
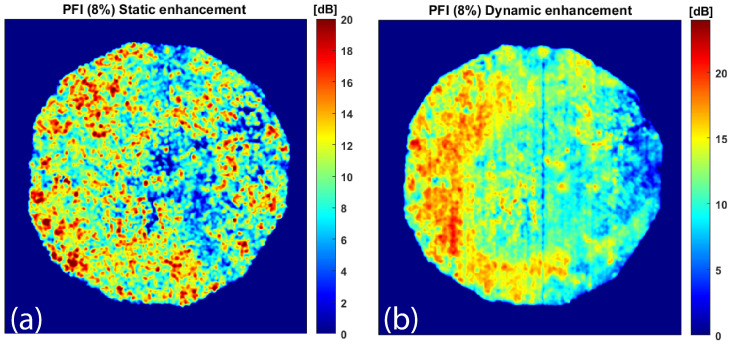
Quantification of signal enhancement produced by partial-field illumination (PFI). (**a**) Static signal enhancement map calculated as the ratio of the static FFOCT signal obtained under partial-field illumination (Figure 4c) to that obtained under full-field illumination (Figure 4a), displayed in decibels (dB). Both images were smoothed using a Gaussian filter prior to division. (**b**) Dynamic signal enhancement map calculated as the ratio of the Phase Fluctuation Index obtained under partial-field illumination (Figure 4f) to that obtained under full-field illumination (Figure 4d), also displayed in dB after Gaussian smoothing. PFI produced a 19.8 dB improvement in static signal and a 23.2 dB improvement in dynamic signal, confirming that reducing incoherent background is particularly beneficial for dynamic functional imaging where useful signals are close to the detector noise floor.

The improvement introduced by PFI was quantified by directly comparing the static and dynamic signal amplitudes (Figure 5).

Static signal enhancement was calculated from the ratio of the conventional 2-phase FFOCT images obtained under PFI and full-field illumination (Figure 5a), while dynamic enhancement was quantified from the ratio of the corresponding PhFI maps (Figure 5b). Prior to division, both images were smoothed using a Gaussian filter with σ = 2 pixels to improve robustness of the comparison.

Quantitative analysis revealed a static signal improvement of 19.8 dB and a dynamic signal improvement of 23.2 dB when using 8% PFI compared with conventional full-field illumination.

The stronger gain observed in dynamic contrast confirms that PFI is particularly beneficial for D-FFOCT functional imaging, where the relevant biological features are often close to the detector noise floor.

Importantly, these gains were obtained while preserving the same reference/sample ratio and without modifying the interferometric detection scheme, demonstrating that PFI acts as a direct sensitivity enhancement strategy rather than a change in image interpretation.

When combined with the ratio-free polarization architecture, PFI provides a cumulative sensitivity improvement of approximately 26.2 dB relative to conventional NPBS-based full-field D-FFOCT. This cumulative value refers to the experimentally measured dynamic sensitivity gain obtained with partial-field illumination (23.2 dB) combined with the independently measured 3 dB ($\sqrt{2}$-fold) gain provided by ratio-free detection. This represents a major improvement for deep functional imaging of retinal organoids and establishes a practical route toward high-sensitivity label-free imaging of complex biological systems under photon-limited conditions.

## 3. Discussion

This study demonstrates that sensitivity optimization in dynamic full-field optical coherence tomography (D-FFOCT) can be substantially improved through two complementary strategies: ratio-free optical flux redistribution and partial-field illumination (PFI). Together, these approaches address the two principal limitations of deep D-FFOCT imaging in highly scattering biological samples: inefficient use of optical power and detector saturation by incoherent background.

The ratio-free polarization architecture addresses part of these limitations by enabling continuous tuning of the sample-to-reference field ratio, thereby allowing optimal optical flux distribution and improved sensitivity. Simultaneously, polarization routing suppresses parasitic reflections, improving detector dynamic range utilization and enabling the use of brighter illumination sources without overwhelming the camera with parasitic background, particularly when operating with sample-dominated splitting ratios. Under ideal source-power conditions and optimal ratio tuning, theoretical sensitivity improvements can exceed 25 dB when optical dose is not the primary constraint.

Although variable splitting strategies have been extensively studied in fiber-based OCT systems, the same principle applies to full-field interferometric imaging. Lippok et al. [39] demonstrated that replacing a fixed 50/50 coupler with a variable-ratio coupler significantly improves the minimum detectable reflectivity by optimizing the balance between sample and reference intensities according to the principle of least detectable reflectivity. Their results support the present ratio-free architecture, where continuous tuning of the sample-to-reference ratio similarly improves detector efficiency and avoids unnecessary loss of useful photon flux. In highly scattering biological samples, where the optimal ratio varies strongly with imaging depth and local reflectivity, this flexibility becomes particularly important for maintaining dynamic sensitivity.

Recent work in dark-field FF-OCT has independently highlighted the importance of adaptive optical flux distribution. Fan et al. demonstrated that adjustable transmission-to-reflection ratios improve both signal-to-noise ratio and imaging depth compared with fixed-ratio architectures, particularly when imaging samples with different scattering properties [35]. Although implemented in a different FF-OCT modality, their results further support the central premise of the present work that optimal sensitivity depends critically on matching the sample-to-reference ratio to the optical properties of the specimen rather than relying on a fixed beam-splitting configuration.

Experimentally in this study, this optical redistribution alone provides a $\sqrt{2}$-fold (3 dB) sensitivity improvement relative to conventional NPBS-based D-FFOCT. Although this gain may appear moderate compared with the larger improvements produced by PFI, it is especially valuable because it is achieved without increasing optical dose, modifying acquisition total time, or altering the biological preparation. It therefore represents a robust baseline improvement that can be directly implemented in existing systems with minimal modification.

Furthermore, the ratio-free architecture offers an important translational advantage for biomedical imaging. For the 810 nm extended LED source used in this study, the ISO/ANSI continuous-wave exposure limit corresponds to 15.8 mW over a 1.5 × 1.5 ${\mathrm{mm}}^{2}$ illuminated area. Under such dose-limited conditions, the ratio-free architecture would be advantageous because the reference intensity can be increased independently of the optical dose delivered to the sample, improving detector dynamic range usage while remaining compatible with optical safety constraints. This feature is particularly important for future in vivo imaging.

Potential polarization-dependent effects should also be considered. In the present free-space Linnik configuration, the short optical path lengths and low-coherence illumination make polarization mode dispersion negligible. Residual birefringence could in principle reduce fringe visibility through polarization mismatch between the interferometer arms, but no measurable degradation of dynamic contrast or system stability attributable to such effects was observed under the experimental conditions used in this study.

Partial-field illumination provides a second and larger improvement by reducing multiply scattered background. Similar contrast improvements were previously demonstrated in retinal imaging by Krafft et al. [36] in an adaptive-optics full-field ophthalmoscope. At 120 μm imaging depth, 8% PFI produced a 19.8 dB improvement in static FFOCT signal and a 23.2 dB improvement in dynamic D-FFOCT sensitivity. Beyond improving imaging depth and dynamic contrast, such sensitivity gains may also relax detector requirements. Since shot-noise-limited sensitivity scales with the square root of the full-well capacity, replacing a 1 ${\mathrm{Me}}^{-}$ detector with a 10 ${\mathrm{ke}}^{-}$ detector would reduce the theoretical SNR by approximately 10-fold, as shown by Equation (3). The 14.5-fold dynamic sensitivity gain obtained with 8% PFI therefore suggests that comparable D-FFOCT sensitivity could, in principle, be achieved using cameras with substantially lower full-well capacity than the 2.3 ${\mathrm{Me}}^{-}$ detector used in the present study. This could facilitate broader adoption of D-FFOCT using more widely available imaging hardware.

The depth of 120 μm was selected because it corresponds to a regime where image quality begins to degrade significantly due to increased background and multiple scattering. This depth therefore provides a particularly relevant test case for evaluating the benefits of partial-field illumination. In principle, the optimal illumination ratio is expected to decrease with increasing imaging depth as incoherent background becomes more significant. However, extending the present analysis to substantially greater depths would have required illumination ratios below the 8% value used here, which could not be reliably achieved with the available iris adjustment range. Furthermore, the resulting illuminated regions become progressively smaller, making it increasingly difficult to identify and quantify comparable biological structures across acquisitions. A systematic characterization of the optimal illumination ratio and sensitivity gain as a function of imaging depth would therefore be valuable future work.

Overall, the improved visualization of retinal organoid cellular organization and intracellular dynamics in Müller glial cell cultures is also consistent with previous demonstrations that dynamic contrast provides functional information beyond conventional structural OCT imaging [24,27,31]. In the present study, cell-type attribution relies on previously characterized retinal organoid models [6] and Müller glial cell models [38], together with morphology and localization, rather than on post-imaging immunofluorescence performed on the same field of view. In particular, the ability to resolve active intracellular compartments, membrane-associated protrusions, highly dynamic filopodia-like extensions, and heterochromatin-like nuclear structures highlights the relevance of D-FFOCT for longitudinal monitoring of organoids and live-cell cultures without exogenous labeling.

The ratio-free architecture also establishes an optimal configuration for interface self-referenced D-FFOCT (iSR-D-FFOCT), where interference occurs between the sample and the substrate interface rather than an external reference mirror. This self-referenced design minimizes sensitivity to mechanical vibrations and phase instability while preserving high dynamic contrast [37]. The improved detection of nuclei, membrane-associated structures, and nanometric interference fringes in Müller glial cells demonstrates the potential of this configuration for probing subtle intracellular dynamics and membrane fluctuations at very high sensitivity.

Such performance is particularly promising for future photostimulation experiments aimed at detecting osmotic responses, membrane deformation, and fine physiological changes associated with ion channel activation. Because these signals are often extremely weak and occur near the detection limit of conventional OCT systems, the combined ratio-free and PFI strategy provides a practical route toward functional imaging of previously inaccessible biological phenomena.

Further reduction in the PFI ratio—potentially down to 0.5% as suggested by related studies [36]—could provide even greater sensitivity gains and stronger evidence that scattering dominates over aberration in these systems. However, this improvement comes at the cost of increased acquisition time, since a smaller illuminated area requires proportionally more image tiles to reconstruct the same total field of view. In the present work, 8% PFI corresponds to approximately a 12.5-fold increase in acquisition time compared with full-field illumination.

While this trade-off may be acceptable for high-sensitivity applications such as photostimulation studies or detailed retinal organoid characterization, it may limit routine large-scale imaging workflows. Future implementations based on structured illumination, sparse illumination arrays, line scanning with descanned detection, or rolling-shutter synchronization could preserve the sensitivity benefits of PFI while reducing acquisition time penalties [40]. In particular, sparse distributed illumination would likely be more efficient than the single-spot implementation used here for suppressing multiply scattered light and, under high-power illumination in the absence of sample dose limitations, could theoretically provide sensitivity improvements approaching 48 dB at 120 μm in a retinal organoid, corresponding to the combined effect of PFI and the additional 25 dB gain predicted for optimal ratio tuning (Figure 2a).

A limitation of the present implementation is that strong PFI reduces the effective field of view and increases acquisition time proportionally. In addition, the optimal sample-to-reference ratio remains sample-dependent and currently requires manual adjustment. Future work will investigate automated ratio optimization and sparse or structured illumination strategies to preserve sensitivity while maintaining imaging throughput.

## 4. Materials and Methods

### 4.1. Optical Setup

The ratio-free D-FFOCT system was built as a polarization-based Linnik interferometer (Figure 1). Illumination was provided by a mounted light-emitting diode (LED; M810L5, Thorlabs, Newton, NJ, USA) centered at 810 nm with a spectral bandwidth of 30 nm, used as an extended spatially incoherent source (S1) with an emitting area of 1 ${\mathrm{mm}}^{2}$. A first lens pair (L1; ACL25416U-B and AC254-150-B-ML, Thorlabs) imaged the source onto the illumination mask (OM). A first polarizer (Pol.1; LPNIRB100-MP2, Thorlabs), combined with a polarizing beam splitter (PBS; CCM1-PBS252/M, Thorlabs), controlled the optical flux ratio between the sample and reference arms by adjusting the balance between horizontal and vertical polarization components.

The illumination mask was relayed to the back focal planes of the two microscope objectives (Obj.1 and Obj.2; UMPLFLN20XW, Olympus/Evident, Japan) using a doublet (L2; AC508-150-B-ML, Thorlabs) and intermediate relay lenses (L3 and L4; AC508-150-B-ML, Thorlabs), forming the Linnik interferometer. A silver mirror (M1; PFE20-P01, Thorlabs) directed the beam into the interferometric arms.

The illumination iris (Ir.1), positioned at the focal plane of L2 and conjugated to the sample plane, was used for partial-field illumination (PFI) to reduce incoherent background generated by multiple scattering. A second iris (Ir.2) was conjugated to the illumination path through the PBS to maintain optical alignment.

Dynamic and static phase shifts were introduced using a piezoelectric actuator (PZT; PK44M3B8P2, Thorlabs) and a linear translation stage (XR50P, Thorlabs), respectively. Zeroth-order quarter-wave plates (QWP1 and QWP2; WPQ10M-808, Thorlabs) rotated the polarization state after reflection from either the sample or the reference mirror, enabling polarization routing through the PBS and strong suppression of parasitic reflections.

A second polarizer (Pol.2) acted as an analyzer to project the orthogonal sample and reference fields onto a common polarization axis before detection by a cMOS camera (Q-2HFW, Adimec, Eindhoven, The Netherlands). A detection mask (DM) was positioned at the focal plane of the detection optics. Steering mirrors (M2, M4, and M5) were used for optical alignment, and the sample was mounted on a motorized 3D translation stage (RAMM RM-1250 and LS-100, ASI, Trevose, PA, USA).

### 4.2. Simulation

The theoretical sensitivity analysis presented in Figure 2 was implemented in MATLAB (R2024a) using the analytical expressions derived from the D-FFOCT signal model. Simulations were based on Equation (2) and the NPBS reference sensitivity expression, using representative parameters corresponding to highly scattering biological samples: $r_{p}^{2}=10^{-3}$, $s^{2}=10^{-7}$, $\gamma =10^{3}$, where $r_{p}^{2}$ represents the effective parasitic optical reflection term, $s^{2}$ is the coherent sample reflectivity, and γ is the ratio between incoherent and coherent sample return. $r_{w/Si}^{2}=0.2$ is the reflectivity of the reference specular surface (water–silicon) used in NPBS-based D-FFOCT. The influence of the polarization angles α and β on the ratio-free architecture was evaluated by calculating the theoretical signal-to-noise ratio (SNR) improvement relative to the conventional NPBS configuration. The MATLAB simulation script used for generating the sensitivity maps and optimization curves is available on GitHub (https://github.com/Tual29/ratio-free-DFFOCT/tree/main (accessed on 11 June 2026). The MATLAB script used for image rendering is available at: https://github.com/noahheldt/A-guide-to-dynamic-OCT-data-analysis/tree/main/HSB%20visualization%20of%20PSD%20moments (accessed on 11 June 2026).

### 4.3. Image Acquisition

Images were acquired using the ratio-free D-FFOCT system shown in Figure 1. Illumination was provided by a mounted light-emitting diode (LED) source centered at 810 nm with a spectral bandwidth of 30 nm, corresponding to a coherence length of 9.62 μm.

The source was coupled to a water-immersion objective (UMPLFLN20XW, Olympus/Evident, Tokyo, Japan) with a numerical aperture of 0.5, resulting in a theoretical transverse resolution of 810 nm and an axial resolution of 3.65 μm [22].

All datasets were acquired over 5.12 s at 100 frames per second (FPS). The polarization angles α and β were adjusted experimentally to maximize SNR under these acquisition conditions. For standard D-FFOCT imaging, both angles were set to $10^{\circ }$. For interface self-referenced D-FFOCT (iSR-D-FFOCT) [37], the optimal self-referenced configuration was obtained by setting $\alpha =90^{\circ }$ and $\beta =0^{\circ }$.

Partial-field illumination (PFI) was implemented using the illumination iris (Ir.1 in Figure 1). For PFI experiments, the illuminated area was reduced to 8% of the full-field configuration while maintaining the same reference-to-sample ratio. Exposure times were adjusted accordingly to preserve comparable detector filling conditions.

### 4.4. Rolling-Phase Detection and Signal Demodulation

Dynamic data were acquired using the rolling-phase modulation regime previously described for D-FFOCT [25]. During acquisition, a continuous linear phase shift equivalent to 0 to 6π was introduced by controlled displacement of the piezoelectric mirror (PZT in Figure 1) throughout the temporal sequence.

This rolling-phase detection avoids phase bias artifacts associated with static interferometric conditions and improves robustness against speckle and fringe artifacts for dynamic structures with limited drift.

Data were demodulated at 585 mHz to isolate the dynamic interferometric contribution and recover temporal fluctuations associated with intracellular activity. For the PFI experiments, 512 raw full-field interferograms were acquired at 100 Hz for both full-field illumination and partial-field illumination conditions.

### 4.5. Two-Phase Imaging (Static Imaging)

To avoid ambiguity in the interpretation of interferometric signal magnitude, 2-phase images were acquired for static signal quantification. Two interferometric images were recorded with a relative phase shift of π between them. Their difference was then calculated and converted to its absolute value, effectively removing the DC contribution and isolating the coherent interferometric component.

For the results shown in Figure 4, a total of 100 2-phase images were acquired and averaged to improve signal robustness and visualization quality.

### 4.6. Dynamic Image Rendering

Three complementary metrics were calculated from the temporal fluctuations of the interferometric signal to characterize cellular dynamics.

The first metric was the recently introduced Phase-Fluctuation-Index (PhFI), defined as $\langle \left|\Delta I(t)\right|\rangle$, which emphasizes the magnitude of local phase fluctuations [25]. This metric is approximately linearly related to both scatterer reflectivity and the distribution of phase variability induced by intracellular active transport. Importantly, it is independent of the initial phase bias of the scatterers and therefore reduces artifacts such as speckle and stationary fringes.

A high PhFI value may correspond either to a strong scatterer with limited motion or to a weak scatterer with large dynamic displacement.

Two additional metrics were computed from the magnitude spectral density (MSD): the mean frequency 〈MSD〉, and its standard deviation StD(MSD) [24].

The mean frequency reflects the characteristic speed of intracellular transport and provides an estimate of cellular activity and motility, whereas the standard deviation helps distinguish directional transport from Brownian motion, thereby separating organized cellular motion from drifting extracellular material.

Together, these three metrics describe:

- Dynamic magnitude through Phase Fluctuation Index (PhFI);
- Quantitative transport speed through mean MSD;
- Motility versus Brownian behavior through MSD standard deviation.

These parameters were assigned to a Hue–Saturation–Brightness (HSB) color space for visualization:

- Hue: mean MSD (〈MSD〉);
- Saturation: standard deviation of MSD (StD(MSD));
- Brightness: PhFI.

In this representation, blue corresponds to slower intracellular dynamics, red to faster transport, low saturation to Brownian motion, and high saturation to directional transport.

Display thresholds were determined using percentile cutoffs of the signal distributions:

- Hue: 0.1% to 99.9%;
- Saturation: 5% to 99.9%;
- Brightness: 5% to 99.9%.

### 4.7. PFI Quantification

In order to quantify the PFI gain in sensitivity ($G_{\mathrm{PFI}}$), the following coefficient was calculated:

(20) $$G_{\mathrm{PFI}}=\frac{{\mathrm{PFI}}_{8\%}-\mathrm{bckg}}{{\mathrm{FF}}_{100\%}-\mathrm{bckg}},$$

where ${\mathrm{PFI}}_{8\%}$ is the pixel value of the PFI image acquired at 8% illumination (either the 2-phase static image or the PhFI image), bckg is the background value for the considered metric when the camera operates in the shot-noise-limited regime, and ${\mathrm{FF}}_{100\%}$ is the pixel value of the standard full-field image acquired under 100% illumination.

### 4.8. Sample Preparation

#### 4.8.1. Retinal Organoids

Human retinal organoids at day 150 of differentiation were maintained in 12-well plastic-bottom culture plates (Corning^®^ Costar^®^ Not Treated Multiple Well Plates, Corning Inc., Corning, NY, USA). Before imaging, each well was filled with pre-warmed fresh culture medium. The differentiation protocol and cellular composition of this retinal organoid model were extensively characterized previously by Reichman et al. in 2017 using immunohistochemistry and molecular analyses, including markers for major retinal cell populations [6].

The organoids were kept in the dark for at least 1 h in a humidified incubator at 37 °C and 5% ${\mathrm{CO}}_{2}$ prior to imaging.

#### 4.8.2. Müller Glial Cells

Human induced pluripotent stem cell-derived Müller glial cells [38] were cultured in glass-bottom dishes precoated with Geltrex^®^.

These cultures were used for interface self-referenced D-FFOCT (iSR-D-FFOCT) imaging to evaluate fine intracellular dynamics and membrane-associated structures under high-sensitivity self-referenced conditions.

## 5. Conclusions

Overall, the cumulative sensitivity improvement of approximately 26.2 dB achieved in this work represents a major step forward for D-FFOCT applied to complex biological systems. More importantly, this improvement is obtained using experimentally accessible modifications that remain compatible with standard high-numerical-aperture FFOCT implementations. Rather than introducing a fundamentally new OCT modality, this work provides a practical framework for optimizing existing D-FFOCT systems for deeper, more sensitive, and more biologically relevant functional imaging.

These results expand the potential of D-FFOCT for retinal research, disease modeling, drug screening, and live-cell monitoring, and support its future translation toward clinical and translational biomedical imaging applications.

## Data Availability

Data and any remaining information can be obtained from the corresponding author upon reasonable request.
